# Comparison of infrarenal aortic balloon occlusion with internal iliac artery balloon occlusion for patients with placenta accreta

**DOI:** 10.1186/s12884-019-2303-x

**Published:** 2019-05-02

**Authors:** Youwen Mei, Hu Zhao, Hui Zhou, Huaibo Jing, Yonghong Lin

**Affiliations:** Department of Obstetrics and Gynecology, Chengdu’s Women and Children’s Central Hospital, Chengdu, Sichuan Province China

**Keywords:** Infrarenal aortic artery balloon occlusion, Internal iliac artery balloon occlusion, Placenta accreta

## Abstract

**Background:**

The study was designed to compare the role of infrarenal aortic artery balloon occlusion (IAABC) with internal iliac artery balloon occlusion (IIABOC).

**Methods:**

One hundred seventy-four cases with placenta accreta were retrospectively analyzed.74 cases who had IAABC were in group A, while the others who had IIABOC were in group B.

**Results:**

Amount of estimated blood loss (EBL), the rate of major blood loss, the rate of blood transfusion and uterine packing, length of hospitalization were not different in both groups. The rate of uterine artery embolization (UAE), balloon occlusion time, operation time and fetus radiation dose in group A were less than those in group B.

**Conclusions:**

IAABC resulted in better clinical outcomes than IIABOC.

## Background

The morbidly adherent placenta is one of the serious complications of pregnancy, resulted in massive hemorrhage, hysterectomy, and perinatal maternal-fetal death [1, 2]. Its incidence has increased rapidly due to the increased rate of cesarean delivery in the past decades [3]. Morbidly adherent placenta could be divided into three types: placenta accreta, increta, and percreta according to invasion depth of placental villi in uterine myometrium [4]. In most published literature, the term “placenta accreta” is used as a general term for all the three types. However, perinatal outcomes vary a lot in these types. In order to increase the study’s accuracy, our study only included patients with placenta accreta, excluding those with placenta increta, and percreta. In particular, placenta accreta refers that placental villi attaches to uterine myometrium or invades superficial uterine myometrium.

Since prophylactic placement of endovascular balloon is introduced in 1979, it has become a popular technique in the treatment of morbidly adherent placenta. There are mainly infrarenal abdominal aorta artery’s, common iliac arteries’ and internal iliac arteries’ balloon according to the balloon’s position [5, 6]. Interventional skills have been applied in our hospital since 2013 and have been applied widely in clinical practice now. According to previous literature, there is no definite conclusion about the comparison between IAABC and IIABOC, therefore, our study is designed to compare their efficacy, in order to provide evidence for obstetrics when dealing with patients with placenta accreta.

## Methods

This was a retrospective observational study conducted in Chengdu women and children’s central hospital. Clinical data from Jan 2013 to Oct 2017 was extracted from the electronic database, after obtaining approval of the Ethics Committee. Written consents for data to be used in scientific research were also acquired from patients before operation.

The inclusion criteria consisted of the following items: diagnosis of placenta accreta, gestational age (GA) > 34 weeks, and prophylactic IAABC and IIABOC performed. The exclusion criteria were emergency cesarean section and abnormal coagulation function. Only cases of placenta accreta were included in our study, not containing placenta increta or percreta. Diagnosis of placenta accreta in our study was made based on the image of ultrasonography (a loss of clear zone and bridging vessels [7]) or MRI (thinning or irregularity of myometrium subjacent to and contiguous with placenta without transmural extension of placenta [8]), and clinical findings (placenta adheres to myometrium but do not invade myometrium) during operation.

Included cases were divided into two groups. Patients who had prophylactic IAABC were in group A, while patients who had prophylactic IIABOC were in group B.A comprehensive evaluation and multidisciplinary treatment plan was made by interventional doctors, anesthetist, obstetrician, and pediatrics in both groups.

### Prophylactic infrarenal abdominal aorta occlusion

**Fig. 1:**
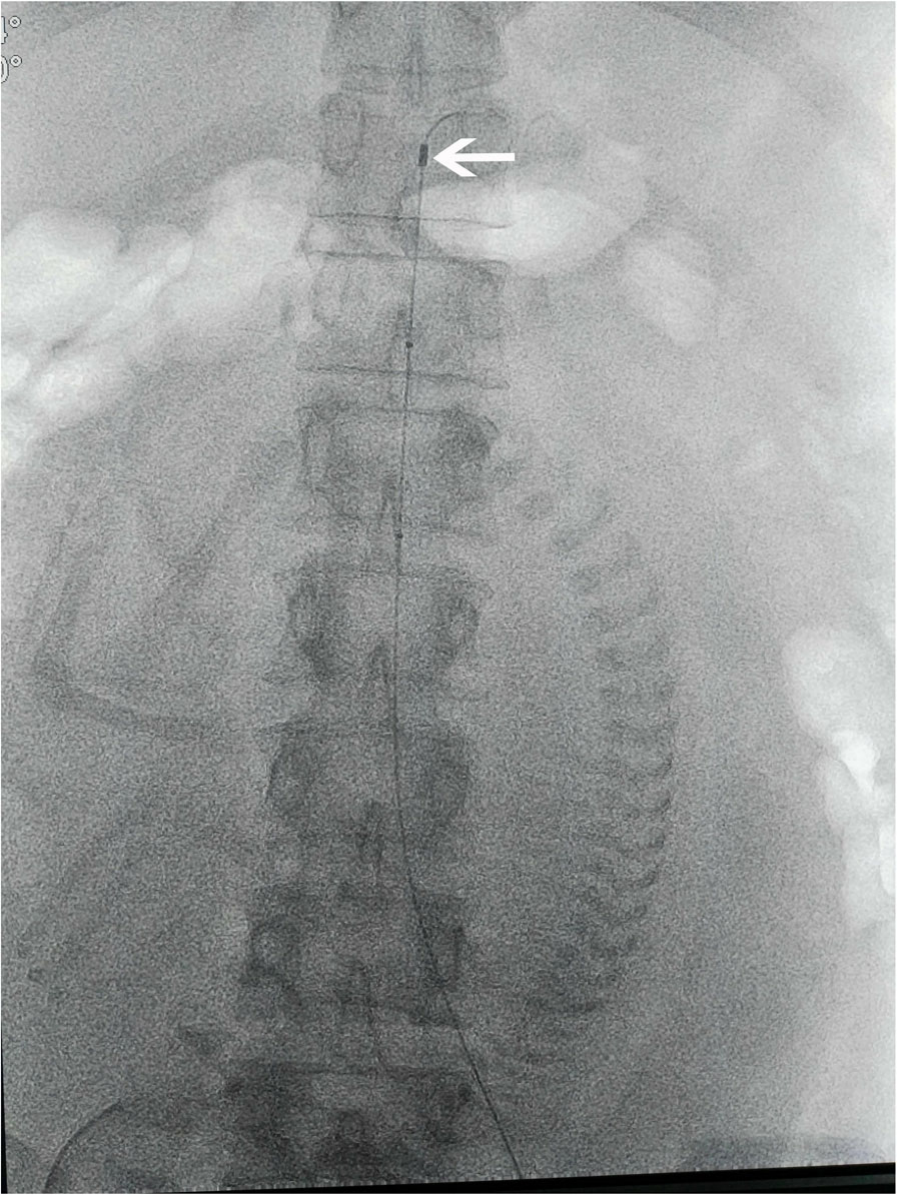
shows that balloon catheter (18*40 mm, PTA, ATLAS) was placed in the aorta with its tip under the level of renal artery

Prior to balloon occlusion, angiography of the abdominal aorta was performed. After local anesthesia, an 8-F sheath was inserted from the right femoral artery using the seldinger technique. Then, A10-F occlusion balloon catheter (18*40 mm, PTA, ATLAS) was placed in the aorta with its tip under the level of the renal artery (Fig. 1). Accurate placement of the balloon was angiographically confirmed with a contrast agent. The balloon was inflated with saline until the patient’s pulse and oxygen saturation on great toe could not be detected. During the cesarean section, the aortic balloon was inflated 5 min and deflated 1 min alternately.

### Prophylactic internal iliac artery occlusion

**Fig. 2:**
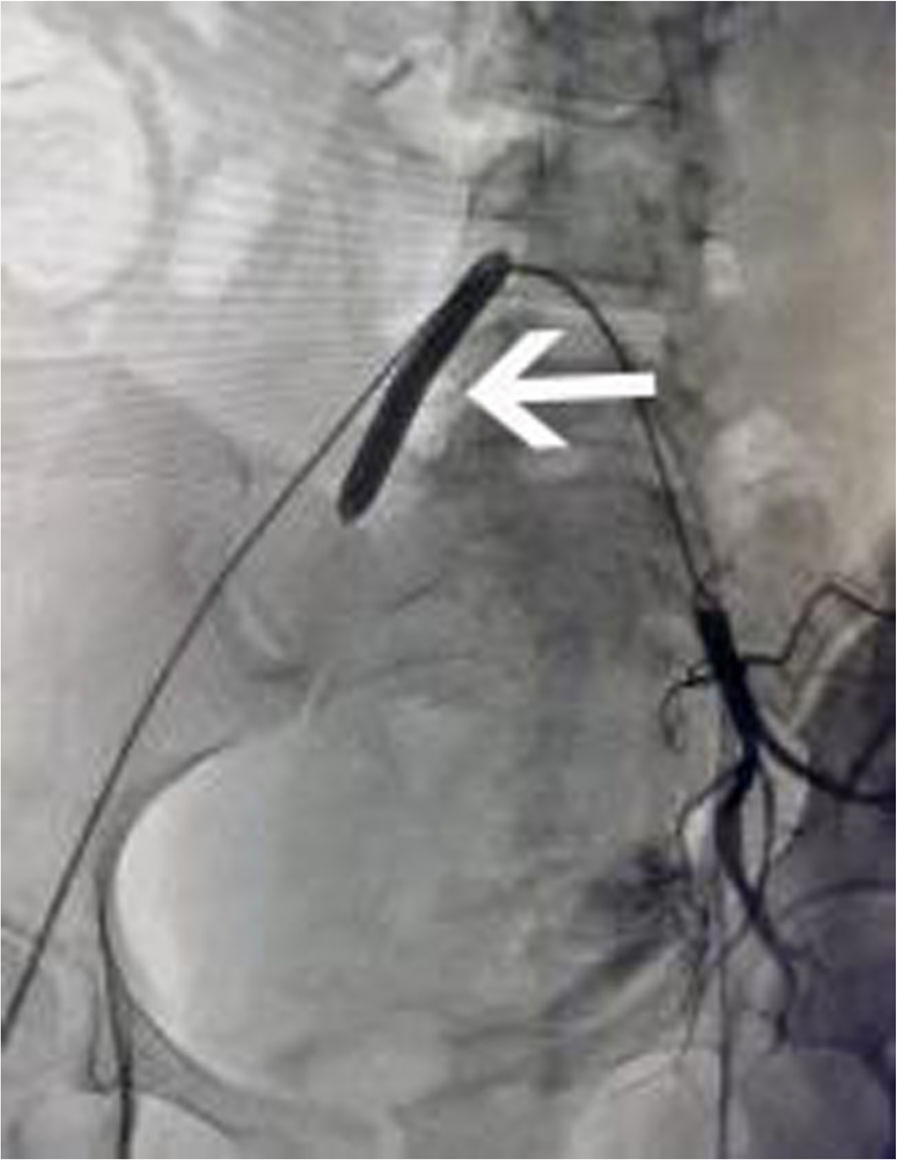
shows balloon of the right internal iliac artery was inflated to block blood flow, while the left uterine artery was thicker and some contrast medium overflowed which indicated it was bleeding after balloon deflated

After local anesthesia, a 6-French sheath was inserted from both femoral arteries. Dual-chamber balloon catheters (8–10 mm*10 cm, PTA-5, cook) were inserted after crossing common iliac artery’s bifurcation. Contrast medium was injected to outline.

the internal iliac arteries. After both balloons were placed in internal iliac arteries (Fig. 2), the balloons were inflated with saline to see if it was effective. During the cesarean section, internal iliac balloons were inflated all the time until hemostasis was attained.

**Fig. 3:**
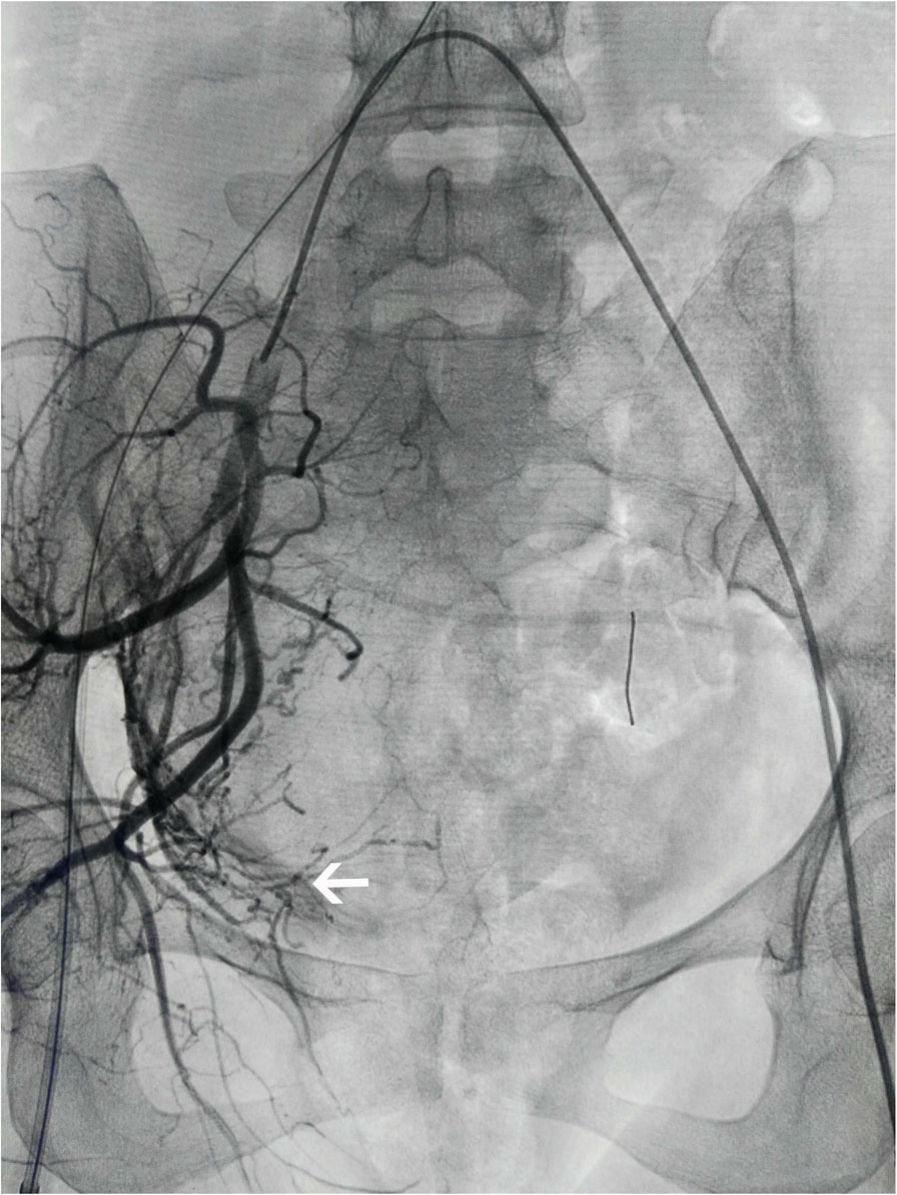
shows that UAE was performed in tortuous and thickened uterine arteries of the right side in angiography

The following procedures were similar in both groups. The placenta was removed manually as far as possible. Conventional skills such as uterine contraction, intrauterine packing (intrauterine packing with ribbon gauze and intrauterine packing with the water-filled balloon), and hysterectomy would be performed if necessary. After obtaining hemostasis, balloons would be extracted. If bleeding continued after skin suture, UAE with absorbable gelatin sponge (710–1000 μm particles, Alicon Pharm, Hangzhou, China) would be performed (Fig. 3). Low molecular weight heparin (LMWH) was given (5000 U, qd *3 days) in both groups.

Blood loss was estimated by weighing surgical pads and by measuring the amount blood loss in the suction jar. Balloon occlusion time was the summation of intermittent time. Major blood loss referred that the volume of blood loss was more than 2000 ml. Postoperative complication mainly included thrombus, fever, and pain. The primary outcome was the amount of EBL, the rate of major blood loss and blood transfusion, occlusion time, radiation dose, operation time, the rate of uterine packing, the rate of UAE and length of hospitalization.

IBM SPSS Statistics for Windows, Version 19.0 was used for data analysis. Shapiro-Wilks test was used to test variables’ normality. Independent T-test or Mann–Whitney U test was used according to variables’ distribution. *P* < 0.05 indicated a significant difference.

## Results

There were 215 patients of placenta accreta from Oct 2013 to Oct 2017 in our hospital. Twenty cases were excluded because they did not receive any interventional skills and 21 cases were excluded because of emergency cesarean section. Finally, there were 174 patients included in our study. Seventy-four cases were assigned in group A, while the others in group B. Prophylactic placement of endovascular balloon were technically successful in all patients. All patients delivered babies by cesarean section.

All variables in both groups were not normally distributed. As a result, the two groups were similar in terms of age, gravidity history, the number of previous cesarean section, the time interval between last cesarean section and this one, rate of complete placenta previa, GA at delivery, neonatal weight and fetus position (Table 1). When it came to outcomes, EBL was not significantly different in both groups [600(300–2500) ml vs 600(300–2500) ml]. The rate of major blood loss, the rate of blood transfusion and uterine packing, and length of hospitalization were also not different in both groups. However, the rate of UAE, balloon occlusion time, operation time and fetus radiation dose in group A were less than those in group B (Table 2). Volume of erythrocyte concentrate transfusion varied from 0 to 6 U in group A, and from 0 to 15.5 U in group B,while volume of fresh frozen plasma varied from 0 to 600 ml in group A, and from 0 to 1400 ml in group B. Five cases suffered fever and ten cases suffered pelvic pain in group A, while seven cases and 12 cases suffered fever and pelvic pain in group B respectively. There was no case of hysterectomy in our study. No organ damage and interventional skills-related complications occurred in both groups.

**Table 1:** Maternal characteristic comparison of both groups

	Age(years old)	Gravidity (times)	Previous cesarean section (times)	Time interval^a^ (years)	Rate of complete placenta previa(%)	Gestational age at delivery (weeks)	Neonatal weight (g)	Rate of fetus head presentation (%)
Group A	31 (21–44)	4 (2–10)	2 (1–3)	6(3–17)	45	36.43(34.86–37.86)	2870(1530–4500)	80
Group B	32 (23–49)	4 (1–9)	2 (1–4)	6(2–18)	36	36.57(34.57–37.43)	2810(2000–4150)	76
P(exact Sig)	0.14	0.21	0.3	0.89	0.28	0.28	0.76	0.58

“a” refers to time interval between last cesarean section and this pregnancy

**Table 2:** Outcomes comparison of both groups

	EBL (ml)	Rate of major blood loss (%)	Rate of blood transfusion (%)	Occlusion time (min)	Radiation dose (mGy)	Operation time (min)	Rate of uterine packing (%)	Rate of uterine packing with water-filled balloon (%)	Rate of UAE (%)	Length of hospitalization (days)
Group A	600(300–2500)	4	15	18(8–29)	1.85 (1–2.9)	40(35–65)	68	46	23	5(2–24)
Group B	600(300–2500)	5	16	25(13–30)	25(18–31)	45(35–75)	60	40	47	5(4–11)
P(exact Sig)	0.316	0.498	0.838	< 0.001	< 0.001	< 0.001	0.34	0.443	0.02	0.652

*EBL* estimated blood loss, *UAE* uterine artery embolization

## Discussion

Intra-operatively, we observed significantly reduced bleeding in the surgical field during occlusion of the aorta/internal iliac artery, thus subjectively confirming the efficacy of our radiological technique. Previous studies also noted that prophylactic IAABC was effective in controlling hemorrhage in placenta accrete [9–12]. As to prophylactic IIABOC, some authors had concluded it was effective in reducing blood loss and rate of hysterectomy in patients with placenta accrete [13–16], while some other literature had opposite conclusions [17–19]. When it came to the comparison of both technique, there were few literatures published. Yan-Li Wang had made a non-randomised prospective study which enrolled 57 patients in IAABC group and 48 cases in IIABOC group. It was found that IAABC had better results with less amount of EBL, less blood transfusion, balloon occlusion time and radiation dose [20]. In our study, IAABC also resulted in better results than IIABOC apart from the amount of EBL. The difference between our study and previous studies may have resulted from different sample and different technique. Our study only included patients with placenta accreta, not containing placenta increta and percreta, but previous studies included patients with placenta accreta, increta, and percreta. In addition, the balloon had a fixed diameter in our study, while the balloons in some previous literature had a variable diameter.

**Fig. 4:**
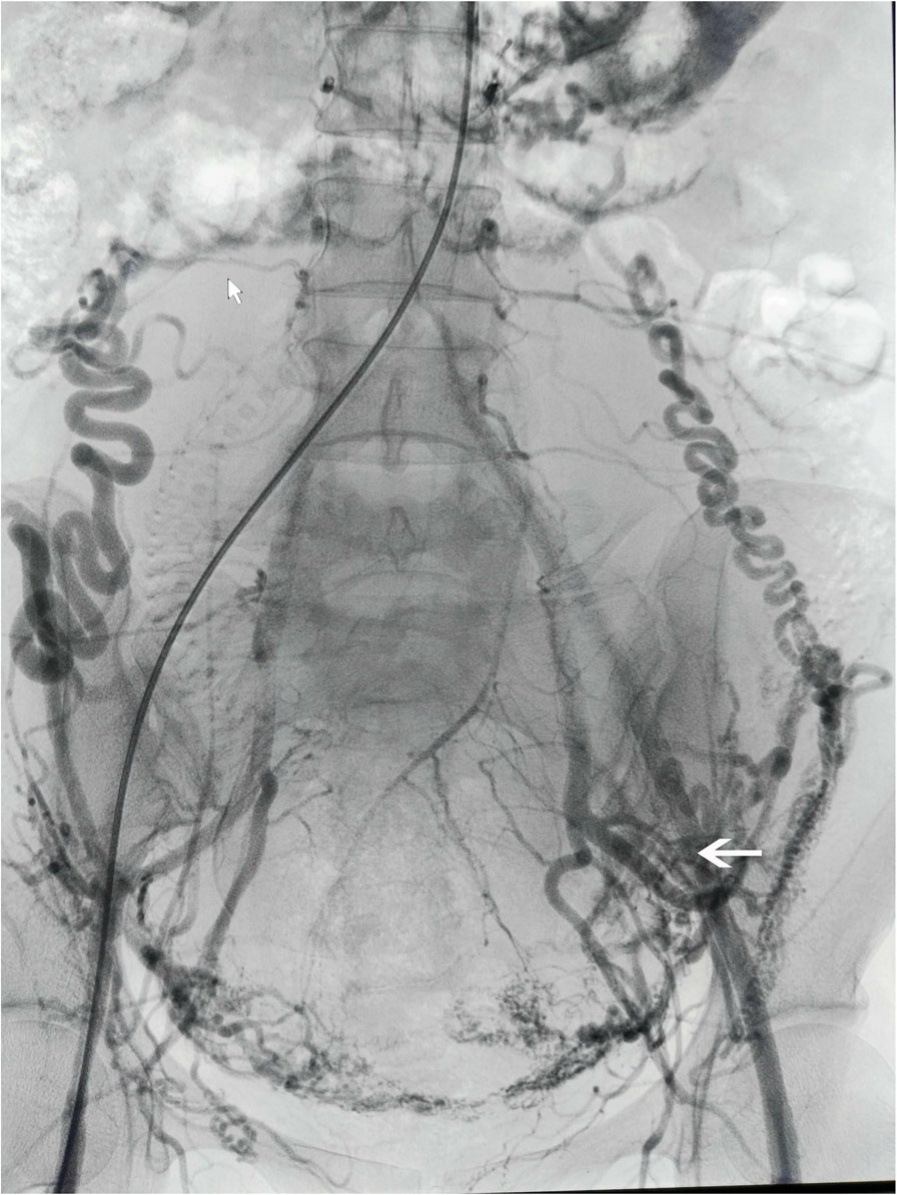
shows bilateral ovarian arteries are interlinked with the uterine arteries and image of collateral vessels

Another interesting result in our study was that the rate of UAE in group A was less than that in group B. The possible explanation may be collateral circulation (Fig. 4) [21]. On one hand, collateral vessels continued to provide blood supply to the pelvic organs after occlusion of internal iliac arteries. On the other hand, abdominal aorta had blocked the blood supply of most collateral vessels. During operation, it was easier for obstetricians to suture bleeding spot or vessel ends in sites where the placenta was attached in a bloodless surgical field. In addition, because occlusion balloon of the aorta was inflated 5 min and deflated 1 min alternately, it was easy to find out bleeding spot, which resulted in a higher rate of thorough hemostasis.

In our study, fetal radiation dose was lower in group A compared to that in group B. In IIABOC, two sets of sheaths and balloons were needed to cross abdominal bifurcation to reach contralateral internal iliac arteries. In contrast, IAABC required only one sheath and balloon insertion, without crossing abdominal bifurcation [21, 22]. Moreover, the aorta is thicker than the inner iliac arteries, which makes it easier to place a balloon.

Occlusion time should be as short as possible to avoid thrombotic complications [23]. There were no interventional skills-related complications in all cases in our study. The reason may be as follows. (1) Balloons in both groups had a fixed size which would not be excessively extended. (2) Occlusion time was relatively short in both groups, both less than 30 min. (3) The saline in balloons was extracted slowly in both groups. (4) LMWH was given for 3 days.

The strengths of our study are as follows. (1) Our hospital is one of the largest special hospitals of obstetrician and gynecology in China which have enough patients with placenta accreta. It is also one of the earliest to introduce intervention skills in the treatment of placenta accreta in China. (2) It only includes patients with placenta accreta which increases the accuracy. (3) The same protocol is performed for all patients, thus reducing potential operator-dependent bias. The limitation of this study includes the following items. Firstly, it is observational and retrospective. Secondly, the definition of placenta accreta in our study differed from that in previous studies, therefore, the comparison between them should be treated cautiously. Thirdly, diagnosis of placenta accreta in our study is based on clinical findings and ultrasound imaging or magnetic resonance imaging (MRI) which may cause selection bias [24].

## Conclusions

In conclusion, prophylactic infrarenal aortic artery balloon occlusion is a better way for patients with placenta accrete than prophylactic internal iliac artery balloon occlusion. However, prospective studies is needed to compare the efficacy and safety of different technologies, and the influence of radiation on fetus should be followed up for a long time.

## Abbreviations

EBL: Estimated blood loss
GA: Gestational age
IAABC: Infrarenal aortic artery balloon occlusion
IIABOC: Internal iliac artery balloon occlusion
LMWH: Low molecular weight heparin
UAE: Uterine artery embolization

## References

[1] Bauer ST, Bonanno C (2009). Abnormal placentation. Semin Perinatol.

[2] Ballas J, Hull AD, Saenz C (2012). Preoperative intravascular balloon catheters and surgical outcomes in pregnancies complicated by placenta accreta: a management paradox. Am J Obstet Gynecol.

[3] Fitzpatrick KE, Sellers S, Spark P (2014). The management and outcomes of placenta accreta, increta, and percreta in the UK: a population-based descriptive study. BJOG Int J Obstet Gynecol.

[4] Jauniaux Eric, Collins Sally L., Jurkovic Davor, Burton Graham J. (2016). Accreta placentation: a systematic review of prenatal ultrasound imaging and grading of villous invasiveness. American Journal of Obstetrics and Gynecology.

[5] Salazar GM, Petrozza JC, Walker TG (2009). Transcatheter endovascular techniques for management of obstetrical and gynecologic emergencies. Tech Vasc Interv Radiol.

[6] Mei J, Wang Y, Zou B (2015). Systematic review of uterus-preserving treatment modalities for the abnormally invasive placenta. J Obstet Gynecol.

[7] Jauniaux Eric, Collins Sally L., Jurkovic Davor, Burton Graham J. (2016). Accreta placentation: a systematic review of prenatal ultrasound imaging and grading of villous invasiveness. American Journal of Obstetrics and Gynecology.

[8] Maldjian C, Adam R, Pelosi M (1999). MRI appearance of placenta percreta and placenta accreta. Magn Reson Imaging.

[9] Wang YLS, Zhang FM, Y H (2017). Aortic balloon occlusion for controlling intraoperative hemorrhage in patients with placenta previa increta/percreta. J Matern Fetal Neonatal Med.

[10] Xie L, Wang Y, Luo FY (2017). Prophylactic use of an infrarenal abdominal aorta balloon catheter in pregnancies complicated by placenta accreta. J Obstet Gynecol.

[11] Wu Q, Liu Z, Zhao X (2016). Outcome of pregnancies after balloon occlusion of the infrarenal abdominal aorta during cesarean in 230 patients with placenta praevia accreta. Cardiovasc Intervent Radiol.

[12] Qiu Z, Hu J, Wu J (2017). Prophylactic temporary abdominal aorta balloon occlusion in women with placenta previa accretism during late gestation. Med.

[13] Alanis M, Hurst BS, Marshburn PB (2006). Conservative management of placenta increta with selective arterial embolization preserves future fertility and results in a favorable outcome in subsequent pregnancies. Fertil Steril.

[14] Tan CH, Tay KH, Sheah K (2007). Perioperative endovascular internal iliac artery occlusion balloon placement in the management of placenta accreta. AJR Am J Roentgenol.

[15] Sivan E, Spira M, Achiron R (2010). Prophylactic pelvic artery catheterization and embolization in women with placenta accreta: can it prevent cesarean hysterectomy?. Am J Perinatol.

[16] Carnevale FC, Kondo MM, de Oliveira Sousa W (2011). Perioperative temporary occlusion of the internal iliac arteries as prophylaxis in cesarean section at risk of hemorrhage in placenta accreta. Cardiovasc Intervent Radiol.

[17] Levine AB, Kuhlman K, Bonn J (1999). Placenta accreta: comparison of cases managed with and without pelvic artery balloon catheters. J Matern Fetal Med.

[18] Shrivastava V, Nageotte M, Major C (2007). Case–control comparison of cesarean hysterectomy with and without prophylactic placement of intravascular balloon catheters for placenta accreta. Am J Obstet Gynecol.

[19] Thon S, McLintic A, Wagner Y (2011). Prophylactic endovascular placement of internal iliac occlusion balloon catheters in parturients with placenta accreta: a retrospective case series. Int J Obstet Anesth.

[20] Wang YL, Duan XH, Han XW (2017). Comparison of temporary abdominal aortic occlusion with internal iliac artery occlusion for patients with placenta accreta- a non-randomised prospective study. Vasa..

[21] Masamoto H, Uehara H, Gibo M (2009). Elective use of aortic balloon occlusion in cesarean hysterectomy for placenta previa percreta. Gynecol Obstet Investig.

[22] Panici PB, Anceschi M, Borgia ML (2012). Intraoperative aorta balloon occlusion: fertility preservation in patients with placenta previa accreta/increta. J Matern Fetal Neonatal Med.

[23] Luo Y, Duan H, Liu W (2013). Clinical evaluation for lower abdominal aorta balloon occluding in the pelvic and sacral tumor resection. J Surg Oncol.

[24] Cahill AG, Beigi R, Heine RP, Silver RM, Wax JR, Society of Gynecologic Oncology; American College of Obstetricians and Gynecologists and the Society for Maternal-Fetal Medicine (2018). Placenta Accreta Spectrum. Am J Obstet Gynecol.

